# Epileptic spasms in individuals with Down syndrome: A review of the current literature

**DOI:** 10.1002/epi4.12412

**Published:** 2020-06-30

**Authors:** Daniel J. Kats, Katherine J. Roche, Brian G. Skotko

**Affiliations:** ^1^ Case Western Reserve University School of Medicine Cleveland OH USA; ^2^ Down Syndrome Program Division of Medical Genetics and Metabolism Department of Pediatrics Massachusetts General Hospital Boston MA USA; ^3^ Department of Pediatrics Harvard Medical School Boston MA USA; ^4^ Harvard‐MIT Division of Health Sciences and Technology Cambridge MA USA

**Keywords:** ACTH, adrenocorticotropic hormone, infantile spasms, trisomy 21, vigabatrin, West syndrome

## Abstract

Epilepsy can occur in individuals with Down syndrome (DS), with epileptic spasms representing the most frequent seizure type in this population. Epileptic spasms can have devastating consequences on the development of individuals with the condition. This review sought to explore the lifetime prevalence and underlying mechanism of epileptic spasms in this population. We also aimed to review the response rate to various treatments, the relapse rate, and the development of subsequent epilepsy or autism in this population. A comprehensive literature search was conducted for articles discussing the lifetime prevalence, diagnosis, treatment, outcomes, or underlying etiology of epileptic spasms in animal models or individuals with DS. According to available literature, the global clinic‐based lifetime prevalence of epilepsy in individuals with DS ranged from 1.6% to 23.1%, with epileptic spasms representing 6.7%‐66.7% of these cases. Response rate to treatment with adrenocorticotropic hormone/corticosteroids was highest (81%) and has the most literature supporting its use, with other regimens, including vigabatrin and other antiepileptic drugs, having lower response rates. Epileptic spasms occur more frequently in children with DS than in the general population, though more studies are needed to determine the true lifetime prevalence of epileptic spasms in this population. Generally, children with DS and epileptic spasms tend to be more responsive to treatment and have better outcomes than children with epileptic spasms of unknown etiology (ie, without DS), in terms of response and relapse rates as well as the development of intractable epilepsy (eg, Lennox‐Gastaut syndrome).

**Key points**

- The global clinic‐based lifetime prevalence of epileptic spasms in individuals with Down syndrome (DS) is 1.8% (95% CI: 1.4%‐2.3%)
- Diagnosis of epileptic spasms tends to be later in children with DS than without DS (mean age 7 months vs. 5 months old, respectively)
- Most patients with DS and epileptic spasms experience developmental delay that is more severe than in those with DS alone
- Response rate of epileptic spasms to adrenocorticotropic hormone was highest (81%) compared to vigabatrin (55%) and other therapies
- There are no currently known predictors of treatment response or developmental outcome

## 1 INTRODUCTION

Down syndrome (DS) is estimated to occur in 12.6 out of every 10 000 live births in the United States.^1^ Epilepsy can occur in individuals with DS, most commonly within the first year of life as epileptic spasms, a condition first described by WJ West in 1841. These ictal flexor or extensor spasms are often seen with a characteristic high‐voltage interictal pattern on electroencephalography (EEG) known as hypsarrhythmia and are accompanied by developmental stagnation or regression.^2^ DS is one of the most common etiologies of epileptic spasms, but the pathogenesis of epileptic spasms in DS is unknown.^3^, ^4^, ^5^, ^6^ It is sometimes attributable to a comorbidity such as hypoxic encephalopathy from congenital heart disease surgery or perinatal asphyxia (these cases are referred to as “structural‐metabolic”).^7^ In other cases, however, the cause of the spasms cannot be easily identified (these cases are referred to as “unknown”) or is attributed to the underlying DS (“genetic” etiology). The consequences of these epileptic spasms can be neurologically devastating for some patients with Down syndrome, so prompt recognition and treatment is necessary to optimize the outcomes of individuals with DS and epileptic spasms.

There have been many case reports and smaller reviews of the literature published about epileptic spasms in individuals with Down syndrome, but there are no recent reviews that compile the available data to gain insight about the lifetime prevalence, current standard of care, and outcomes of individuals with this condition.^8^, ^9^, ^10^, ^11^ This comprehensive review of the available literature aims for several objectives: to provide an updated understanding of the pathogenesis of epileptic spasms; to describe the clinical characteristics and diagnosis of the disorder in this population; and to discuss the treatment response and developmental outcomes in patients with DS and epileptic spasms.

## 2 METHODS

### 2.1 Sources

A comprehensive literature search of the PubMed, MEDLINE, Embase, and Web of Science databases was conducted on April 25, 2020 (Table 1). We also searched the references of original articles obtained via these initial searches for additional publications to include in the review. References were managed using Zotero (Corporation for Digital Scholarship, Vienna, VA and Roy Rosenzweig, Center for History and New Media, Fairfax, VA).

**TABLE 1:** Electronic search strategy

1	(“down syndrome”[MeSH Terms] OR “down syndrome”[All Fields]) AND (“spasms, epileptic”[MeSH Terms] OR “epileptic spasms”[All Fields])
2	(“trisomy 21”[MeSH Terms] OR “trisomy 21”[All Fields] OR “down's syndrome”[All Fields]) AND (“West syndrome”[MeSH Terms] OR “West syndrome”[All Fields])
3	1 and 2
4	“hypsarrhythmia”[All Fields] OR “epileptic syndromes”[MeSH terms] OR “nodding spasm”[All Fields]
5	3 and 4

### 2.2 Study selection

We screened titles and abstracts to identify papers pertaining to epileptic spasms or West syndrome, as well as Down syndrome. The full‐length versions of those papers identified in the initial step were obtained and subsequently screened for inclusion in the literature review. Inclusion criteria were as follows: (a) full‐length article was in English and (b) patients described did not have epileptic spasms due to a known structural lesion or other underlying cause (ie, not “structural‐metabolic” epileptic spasms). Articles must have discussed epileptic spasms in individuals with or animal models of DS and/or the lifetime prevalence, diagnosis, treatment, outcomes, or underlying etiology of epileptic spasms in the DS population. No restriction was placed on the year of publication of the articles.

### 2.3 Data analysis

To analyze the lifetime prevalence of epileptic spasms and/or epilepsy in patients with Down syndrome as reported in prior studies, data from several papers were aggregated independently by the first two authors to ensure correct interpretation/transcription. Discrepancies were discussed and resolved to mutual satisfaction by further review of the data. Means and 95% confidence intervals were calculated using IBM SPSS Statistics (IBM Corp).

## 3 RESULTS AND DISCUSSION

### 3.1 Lifetime prevalence

Though the true lifetime prevalences of epileptic spasms and/or epilepsy in DS are not known due to a lack of population‐based studies, several articles have been published reporting patient numbers from various clinics and medical centers worldwide. The global clinic‐based lifetime prevalence of epileptic spasms in individuals with DS ranged from 0.4% to 12.8%, with a median of 2.1% (Table 2).^5^, ^11^, ^12^, ^13^, ^14^, ^15^, ^16^, ^17^, ^18^, ^19^, ^20^, ^21^ The lifetime prevalence from the aggregate data is 1.8% (95% CI 1.4%‐2.3%). While the range of prevalences is wide from the data, 1.8% is within the 95% CI of 7 out of the 12 studies. In the general population, the lifetime prevalence of epileptic spasms is about 0.02%‐0.035%.^22^

**TABLE 2:** Reported lifetime prevalence of epileptic spasms (ES) in individuals with Down syndrome

Paper (Year)	Number of subjects	Subjects with ES	Percentage with ES (95% CI)	Age of onset	
				<1 year of age	>1 year of age
Barca (2014)^12^	39	5	12.8% (2.3%‐23.3%)	5	0
Meeus (2015)^21^	130	8	6.2% (2.0%‐10.3%)	8	0
Romano (1990)^15^	113	4	3.5% (0.1%‐6.9%)	4	0
van Trotsenburg (2006)^19^	176	6	3.4% (0.7%‐6.1%)	6	0
Escofet (1995)^53^	286	9	3.1% (1.1%‐5.2%)	^a^	^a^
Goldberg‐Stern (2001)^13^	350	9	2.6% (0.9%‐4.2%)	9	0
Pueschel (1991)^14^	405	6	1.5% (0.3%‐2.7%)	6	0
Yamanouchi (2005)^5^	71	1	1.4% (0.0%‐4.1%)	0	1
Tatsuno (1984)^18^	844	8	0.9% (0.3%‐1.6%)	7	1
Stafstrom (1991)^17^	737	6	0.8% (0.2%‐1.5%)	^a^	^a^
Kwong (1996)^20^	124	1	0.8% (0.0%‐2.4%)	^a^	^a^
Śmigielska‐Kuzia (2009)^16^	252	1	0.4% (0.0%‐1.2%)	0	1
Overall	3527	64	1.8% (1.4%‐2.3%)		

^a^ Specific age of onset unavailable for some patients.

The clinic‐based lifetime prevalence of epilepsy in DS ranges from 1.6% to 23.1%, with a median of 7.5% (Table 3).^5^, ^12^, ^13^, ^14^, ^15^, ^16^, ^17^, ^18^, ^20^, ^21^ The lifetime prevalence from the aggregate data is 5.8% (95% CI 5.0%‐6.7%). While the range of prevalences among the studies is wide, 5.8% is within the 95% CI of 7 out of the 10 studies. The general population has a 0.6% prevalence of active epilepsy.^23^

**TABLE 3:** Reported lifetime prevalence of epilepsy in individuals with Down syndrome

Paper (Year)	Number of subjects	Subjects with epilepsy	Percentage with epilepsy (95% CI)	Age of onset	
				<1 year of age	>1 year of age
Barca (2014)^12^	39	9	23.1% (9.9%‐36.3%)	5	4
Meeus (2015)^21^	130	12	9.2% (4.3%‐14.2%)	9	3
Romano (1990)^15^	113	9	8.0% (3.0%‐13.0%)	5	4
Pueschel (1991)^14^	405	33	8.1% (5.5%‐10.8%)	10	15
Goldberg‐Stern (2001)^13^	350	28	8.0% (5.2%‐10.8%)	^a^	^a^
Yamanouchi (2005)^5^	71	5	7.0% (1.1%‐13.0%)	0	5
Stafstrom (1991)^17^	737	47	6.4% (4.6%‐8.1%)	19	28
Śmigielska‐Kuzia (2009)^16^	252	15	6.0% (3.0%‐8.9%)	2	13
Tatsuno (1984)^18^	844	19	2.3% (1.3%‐3.3%)	11	8
Kwong (1996)^20^	124	2	1.6% (0.0%‐3.8%)	^a^	^a^
Overall	3065	179	5.8% (5.0%‐6.7%)		

^a^ Specific age of onset unavailable for some patients.

The clinic‐based lifetime prevalence of epileptic spasms in patients with known seizures and DS ranges from 6.7% to 66.7%, with a median of 37.1% (Table 4).^4^, ^5^, ^12^, ^13^, ^14^, ^15^, ^16^, ^17^, ^18^, ^20^, ^21^, ^24^ The lifetime prevalence from the aggregate data is 37.4% (95% CI 31.9%‐42.9%). While the range of prevalences is wide from the data, 37.4% is within the 95% CI of 8 out of the 12 studies.

**TABLE 4:** Reported percentage of patients with epileptic spasms (ES) out of patients with known Down syndrome + seizures

Paper (year)	Number of subjects	Subjects with ES	Percentage with ES (95% CI)	Age of onset	
				<1 year of age	>1 year of age
Meeus (2015)^21^	12	8	66.7% (40.0%‐93.3%)	8	0
Barca (2014)^12^	9	5	55.6% (23.1%‐88.0%)	5	0
Kumada (2005)^4^	19	10	52.6% (30.2%‐75.1%)	10	0
Kwong (1996)^20^	2	1	50.0% (0.0%‐100.0%)	^a^	^a^
Verrotti (2013)^24^	104	51	49.0% (39.4%‐58.6%)	48	3
Tatsuno (1984)^18^	19	8	42.1% (19.9%‐64.3%)	7	1
Goldberg‐Stern (2001)^13^	28	9	32.1% (14.8%‐49.4%)	9	0
Romano (1990)^15^	9	4	44.4% (12.0%‐76.9%)	4	0
Pueschel (1991)^14^	25	6	24.0% (7.3%‐40.7%)	6	0
Yamanouchi (2005)^5^	5	1	20.0% (0.0%‐55.1%)	0	1
Stafstrom (1991)^17^	47	6	12.8% (3.2%‐22.3%)	^a^	^a^
Śmigielska‐Kuzia (2009)^16^	15	1	6.7% (0.0%‐19.3%)	0	1
Overall	294	110	37.4% (31.9%‐42.9%)		

^a^ Specific age of onset unavailable for some patients.

While there is a wide variability in these estimates across studies, most studies have similar calculated prevalences and support our aggregate calculations, with a few outliers. The low prevalence of epileptic spasms greatly limits the precision with which individual studies are able to determine lifetime prevalence. It is also likely that epileptic spasms are more prevalent in clinics/hospitals than in the general population of people with Down syndrome. While it is possible that there is geographical variation or other sources of error, there are not enough data to assess those possibilities.

Only three published studies have reported gender demographics on patients with DS and epileptic spasms, so there is not enough data to assess the gender distribution of epileptic spasms within DS.^13^, ^18^, ^25^

### 3.2 Pathogenesis

There are several animal models and investigated cellular pathways of epileptic spasms that are out of the scope of this review, but they are covered in‐depth in several other reviews.^26^, ^27^, ^28^, ^29^, ^30^, ^31^

#### 3.2.1 Animal models

There are several criteria for the ideal animal model of epileptic spasms: (a) Seizures must be specific to the epileptic stage; (b) seizures must be spontaneous, clustered, and spasm‐like (flexor, extensor, or mixed); (c) interictal EEG must resemble hypsarrhythmia and ictal EEG must demonstrate electrodecremental response (EDR); (d) there must be developmental regression or stagnation; and (e) there must be epileptic spasms‐like response to antiepileptic drugs (AEDs).^31^, ^32^

The Ts65Dn mouse model of DS satisfies several of these criteria without any additional modification.^33^ While there are no spontaneous seizures at baseline, extensor spasms and EDR can be induced by administration of baclofen (a GABA_B_ agonist) or γ‐butyrolactone (a prodrug of the GABA_B_ agonist γ‐hydroxybutyrate) to Ts65Dn mice between one week and two months of age.^32^ Furthermore, these induced spasms are prevented/aborted with vigabatrin, ACTH, valproate, and ethosuximide, but are exacerbated by 5‐hydroxytryptophan (a serotonin precursor).^32^ GABA_B_ receptor‐2 expression was also significantly increased in the thalamus and medulla oblongata of these mice, compared to controls.^32^ We discuss the specific cellular pathways for seizures in Ts65Dn mice in the following section, *Cellular Pathways*. The main limitations with the Ts65Dn model are that the seizures are not restricted to the epileptic stage and are not spontaneous.^26^

In addition to congenital models, there are several acquired models of epileptic spasms. For example, an intracerebrovascular injection of corticotropin‐releasing hormone (CRH) during the second week of life in rats will inhibit the hypothalamic‐pituitary‐adrenal (HPA) axis, leading to severe seizures (“the CRH model”).^34^ The associated EEG demonstrates rhythmic and sharp activity, but there are no epileptic spasms. Administration of adrenocorticotropic hormone (ACTH) does not have an effect on these seizures.

Intraperitoneal injection of *N*‐methyl‐d‐aspartate (NMDA) in rat pups between postnatal days five and ten leads to clusters of acute seizures with hyperflexion and tonic spasms of the entire body (“the NMDA model”).^35^ The interictal EEG shows slow waves with superimposed fast activity, which, while not true hypsarrhythmia, is similar. Later in adulthood, there are cognitive, spatial, and memory deficits.^36^ If rats are prenatally subjected to betamethasone (mimicking prenatal stress and altering the HPA axis) or are exposed to restraint stress, spasms start earlier and are more frequent.^37^, ^38^, ^39^ Pretreatment with ACTH or methylprednisolone leads to reduced frequency of spasms and increased latency of onset. Similarly, pretreatment with vigabatrin suppresses spasms.^37^ Pretreatment with ganaxolone (a steroid and GABA_A_ positive modulator) also delays onset of spasms.^40^

#### 3.2.2 Cellular pathways

Specific human genes involved in the pathogenesis of epileptic spasms have been difficult to identify because of small sample sizes. In a study of children with epileptic spasms and co‐occurring DS or tuberous sclerosis complex, most pathogenic genes were only seen in one or two patients.^41^

The G protein‐regulated inward‐rectifier potassium channel 2 (GIRK2) protein, which is linked to the GABA_B_ receptor in neurons, has been implicated in epileptic spasms and DS from studies of the Ts65Dn mouse model. The *GIRK2* gene is part of the region that is triplicated in the mice (and humans with DS). Detriplicating the gene leads to return of normal EEG activity and lack of seizures in Ts65Dn mice.^42^ Similarly, administering tertiapin‐Q (a GIRK antagonist) to Ts65Dn mice also rescued them from spasms and EEG abnormalities.^42^ In the detriplicated model, GIRK2 levels were reduced in the cortex, thalamus, hippocampus, and brainstem relative to the Ts65Dn mice (and comparable to the levels in wild‐type mice).^42^ However, isolated triplication of *GIRK2* in wild‐type mice did not lead to spasms or abnormal EEG findings, suggesting that *GIRK2* overexpression is necessary, but not sufficient, for epileptic spasms.^43^

While vigabatrin inhibits GABA transaminase, indirectly stimulating GABA_B_ receptors (similar to the action of *GIRK2*), as well as GABA_A_ receptors, it paradoxically treats epileptic spasms, suggesting a different pharmacologic mechanism of action.^42^ One possible explanation is the “mammalian target of rapamycin” (mTOR) hypothesis. A serine/threonine kinase, mTOR, is implicated in a variety of cellular functions, such as metabolism, inflammation, hormonal signaling, and cell survival. It also plays a role in neuronal development and function, although its specific functions are not yet well‐defined. In tuberous sclerosis complex, a condition with excessive mTOR activity, one‐third of the epilepsy cases manifest as epileptic spasms, suggesting that mTOR plays a role in the pathophysiology of the disease.^44^ It was found that vigabatrin treats seizures and partially inhibits mTOR in mouse models of tuberous sclerosis complex.^45^ In the multiple‐hit model of epileptic spasms, the mTOR pathway was found to be overactivated in the cortical regions of pups during spasms.^46^ Rapamycin (an mTOR inhibitor) administration led to a dose‐dependent suppression of spasms and improvement of learning deficits in the rats.^46^ Pretreatment with rapamycin in the NMDA model did not lead to any noticeable effect on NMDA spasms.^37^

### 3.3 Diagnostic process

Time from onset of epileptic spasms to diagnosis in children with DS has been found to be delayed by two main factors. First, epileptic spasms can be mistaken for a variety of other medical conditions that frequently co‐occur with DS—such as hypotonia, developmental delay, or gastroesophageal reflux disease.^47^, ^48^ Secondly, the signs of epileptic spasms can be subtle, leading to delay between the onset of symptoms and the caregivers becoming sufficiently concerned to seek medical care.^49^ As of publication, no studies have examined interventions to reduce delay in diagnosis by either of these causes, which should be a high priority for future research. In one study of 12 children, only two patients had less than one month between onset and diagnosis (range for detection: 2 weeks to 25 months).^49^

While the signs and symptoms of epileptic spasms are similar among children with and without DS, age of onset is different. Children with DS tend to experience onset later than children without DS (mean age 7 months old vs 5 months old, respectively).^50^

There are mixed reports regarding potential developmental delay prior to onset of epileptic spasms (compared to children with DS that do not develop epileptic spasms). However, the majority of patients do experience developmental delay relative to their peers with DS once epileptic spasms begin.^25^, ^51^, ^52^ This further delay can become clinically significant before the epileptic spasms are diagnosed.^5^

### 3.4 Electrophysiological and imaging findings

The majority of children with DS and epileptic spasms exhibit hypsarrhythmia on EEG, but modified hypsarrhythmia is also prevalent.^4^, ^8^, ^10^, ^11^, ^12^, ^13^, ^15^, ^18^, ^24^, ^25^, ^48^, ^49^, ^50^, ^51^, ^52^, ^53^, ^54^, ^55^ The presence of either hypsarrhythmia or modified hypsarrhythmia, however, is not prognostically valuable. In the aggregation of published patients with documented EEG and outcome (n = 141), 94/126 (75%) of patients with hypsarrhythmia and 11/18 (61%) of patients with modified hypsarrhythmia achieved complete control of epileptic spasms (*P* = .23).

Computed tomography (CT) of the head is frequently normal in patients with DS and epileptic spasms, but volume loss is also a common finding.^10^, ^15^, ^17^, ^18^, ^25^, ^51^, ^52^, ^53^ Magnetic resonance imaging (MRI) of the head is commonly abnormal—of the 49 patients with MRI results published in the literature, 24 had abnormal findings—but no specific finding has been identified to be prevalent within this group.^5^, ^17^, ^20^, ^24^, ^25^, ^54^ One retrospective review of 36 children with Down syndrome and epileptic spasms found that patients with structural abnormalities on MRI were more likely to have refractory epilepsy than the patients without them.^57^ While these abnormalities did not influence medical treatment, they could potentially be studied for utility in prognostication. Unlike CT and MRI, positron emission tomography (PET) frequently reveals metabolic abnormalities in the brains of people with epileptic spasms (42/140 abnormalities with CT and MRI vs 134/140 with PET).^58^ However, the overwhelming majority of patients in this study did not have DS, so the applicability to the DS population is unclear. To date, no publications have examined PET imaging in patients with DS and epileptic spasms. Since many of these patients develop epilepsy, it may be worthwhile to examine the relationship between metabolically active regions of the brain during epileptic spasms and future epileptic foci in the hopes of risk‐stratifying patients with respect to risk of developing epilepsy. Future research should also examine potential screening modalities for epileptic spasms, since their manifestations can be subtle.

### 3.5 Treatment

Table 5 shows the aggregated results of therapy with ACTH/corticosteroids versus vigabatrin, and Table 6 shows the results of other AEDs and combination therapy for epileptic spasms in the DS population.^10^, ^11^, ^13^, ^18^, ^21^, ^25^, ^48^, ^49^, ^50^, ^51^, ^52^, ^54^, ^55^, ^56^ Overall, the treatment responses for children with DS and epileptic spasms appear to mimic the responses for the general population of children with epileptic spasms (regardless of DS). Like in the general population, ACTH/corticosteroids and vigabatrin are the mainstays of therapy. ACTH/corticosteroids have the strongest evidence supporting their use in the DS population, with 85 subjects identified for whom results of therapy were reported. Response rate (81%) was high, while relapse rate (30%) was relatively low. Vigabatrin had a lower response rate (55%) with a lower relapse rate (25%), but the combined sample size for this monotherapy was much smaller (22 patients), and the response rate varied greatly among studies. Combination therapy had a similar response rate (74%), while an aggregate of all other AEDs had a much lower one (31%). Preference is usually given to ACTH/corticosteroids over vigabatrin due to generally milder side effects/toxicity (the side effect profiles of both medications in patients with DS are comparable to those in the general population).

**TABLE 5:** Treatment outcomes of epileptic spasms in individuals with Down syndrome

	Number of subjects	ACTH/Corticosteroids monotherapy			Vigabatrin monotherapy		
		# Subjects on therapy	Response	Relapse	# Subjects on therapy	Response	Relapse
Pollack (1978)^10^	5	2	100%	0%	‐	‐	‐
Tatsuno (1984)^18^	8	5	100%	40%	‐	‐	‐
Stafstrom (1994)^11^	17	13	92%	‐	‐	‐	‐
Silva (1996)^25^	14	10	100%	40%	2	100%	50%
Goldberg‐Stern (2001)^13^	9	2	100%	50%	‐	‐	‐
Nabbout (2001)^52^	5	‐	‐	‐	5	80%	0%
Eisermann (2003)^51^	18	7	100%	‐	4	100%	‐
Karvelas (2009)^56^	4	3	67%	33%	2	50%	0%
Sanmaneechai (2013)^49^	12	10	70%	57%	‐	‐	‐
Tapp (2015)^48^	13	2	100%	0%	‐	‐	‐
Marandi (2016)^50^	15	9	22%	0%	5	0%	‐
Beatty (2017)^55^	11	11	100%	0%	‐	‐	‐
Armstrong (2019)^54^	21	11	64%	27%	4	25%	25%
Overall	152	85	81%	30%	22	55%	25%

**TABLE 6:** Treatment outcomes of epileptic spasms in individuals with Down syndrome

	Number of subjects	Other AEDs monotherapy			Combination therapy		
		# Subjects on therapy	Response	Relapse	# Subjects on therapy	Response	Relapse
Pollack (1978)^10^	5	‐	‐	‐	3	33%	0%
Tatsuno (1984)^18^	8	1	100%	0%	2	50%	0%
Stafstrom (1994)^11^	17	6	67%	0%	3	33%	0%
Silva (1996)^25^	14	3	67%	0%	‐	‐	‐
Goldberg‐Stern (2001)^13^	9	7	86%	0%	2	100%	0%
Nabbout (2001)^52^	5	‐	‐	‐	1	0%	0%
Eisermann (2003)^51^	18	1	100%	‐	6	83%	‐
Sanmaneechai (2013)^49^	12	6	17%	0%	1	0%	0%
Meeus (2015)^21^	8	‐	‐	‐	8	88%	0%
Tapp (2015)^48^	13	1	100%	0%	10	90%	0%
Marandi (2016)^50^	15	36	11%	0%	2	100%	0%
Armstrong (2019)^54^	21	6	17%	0%	‐	‐	‐
Overall	145	67	31%	0%	38	74%	0%

A ketogenic diet did demonstrate some success at controlling refractory epileptic spasms in patients with DS, but the sample size in the literature is small (10 reports).^50^, ^59^, ^60^ Overall, 8 out of 10 patients responded to the treatment, with half of them achieving complete seizure control. In the general population of patients with epileptic spasms (but not necessarily DS), ketogenic diet is reasonably efficacious for reducing the severity of seizures in patients resistant to first‐line therapy (ACTH/corticosteroids and vigabatrin).^61^, ^62^ While generally well tolerated, adverse effects include transient vomiting, lethargy, refusal to eat or drink, and diarrhea; a study of 104 infants (mostly without DS) with epileptic spasms treated with ketogenic diet reported adverse effects in one‐third of their sample.^60^, ^61^ Unfortunately, the body of research on children with DS and epileptic spasms treated with ketogenic diet is small, so more research is necessary to show safety and efficacy in this population.

Vitamin B_6_ has been used as treatment for epileptic spasms, but there is scant literature on its use as a monotherapy. Only one report was found of successful treatment without combination with prednisone or ACTH.^25^ There are four documented cases of improvement, but they all involve combination with either ACTH or prednisone.^10^, ^63^ Similarly, phenobarbital has only been documented as effective in two cases when it was used in combination with prednisone.^10^ It was unsuccessful in three cases when used without prednisone.^10^, ^53^ In one instance, it did successfully abort epileptic spasms, but relapse was experienced 16 months later and required valproate and hydrocortisone for control.^8^

### 3.6 Outcomes

Post‐treatment EEG normalized in the majority of reported cases that did not progress to epilepsy.^12^, ^24^, ^64^

Predicting prognosis for patients is important for planning treatment and counseling parents; however, there are no currently known predictors of treatment response. Some have suggested that shorter delays between onset of spasms and initiation of treatment lead to better outcomes (developmentally and neurologically), but data are limited and more research is required.^25^, ^51^, ^52^ There is a strong correlation between poor seizure outcome and severe intellectual disability (as measured by IQ), but causation has not been shown.^4^

Generally, children with DS and epileptic spasms tend to be more responsive to treatment and have better outcomes than children with epileptic spasms of unknown etiology (ie, without DS), in terms of response and relapse rates.^51^, ^55^ However, time between initiation of treatment and response did not differ between the two groups.^55^

### 3.7 Developmental implications

Developmental delay (relative to individuals with DS without epileptic spasms) commonly develops after the onset of epileptic spasms. Even if the epileptic spasms are treated sufficiently to achieve long‐term remission, there is frequently a long‐lasting negative developmental impact.^12^, ^13^, ^19^, ^48^, ^49^, ^51^, ^53^, ^54^, ^65^ Behavior and cognition tend to be affected more than motor development.^5^, ^11^ Few studies have examined the lifetime prevalence of autism spectrum disorder (or its associated features) in the population of people with DS and epileptic spasms, but there has been a correlation noted between the two.^13^, ^49^, ^51^

While a developmental delay frequently develops during the course of epileptic spasms, many studies have noted that the delay either improves or resolves altogether after cessation of spasms.^12^, ^25^, ^47^, ^52^ The effect of shorter time between onset and treatment on progression of development is unclear, as few studies have examined this and have conflicting results.^48^, ^51^

## 4 CONCLUSIONS

Although DS is classically associated with the neurological features of intellectual disability and hypotonia, individuals with DS can also develop seizures, including epileptic spasms that can have a profound, and often devastating, impact on a patient's development and future outcomes. This study aimed to review all the relevant literature pertaining to epileptic spasms in DS in order to better characterize the current knowledge of underlying etiology, clinical and EEG features, response to treatment, and outcomes of individuals in this population.

Many studies have explored the lifetime prevalence of epileptic spasms in various clinic‐based cohorts of patients with DS, but a population‐based study would be required to determine the true lifetime prevalence of epileptic spasms. At this time, no population‐based databases are available, although efforts are underway.^66^ Additionally, further studies are required to determine the gender differences in incidence and time to diagnosis, as well as the impact of gender on treatment outcome and prognosis, as these remain unclear. The reported lifetime prevalence in all the published studies, however, exceeds that of the general population, consistent with previous studies demonstrating an increased frequency of epileptic spasms and epilepsy among individuals with DS.^67^

Analysis of treatment outcomes in patients with DS and epileptic spasms, however, suggests that individuals with DS have a more favorable response to treatment than patients with epileptic spasms due to other causes, with many studies demonstrating a high rate of response to ACTH as well as vigabatrin. These comparisons are limited, however, due to lack of consistency of dosing across studies, as well as variable follow‐up periods. Though it has been suggested that a shorter time between the onset of epileptic spasms and initiation of treatment leads to better treatment outcomes, there is limited and conflicting literature on this topic, and this remains an area ripe for further study. Several studies have shown better outcomes in patients with DS and epileptic spasms when compared to other causes of epileptic spasms, with fewer patients going on to develop persistent seizures or other forms of epilepsy, such as Lennox‐Gastaut syndrome.

Though several mouse models of epileptic spasms exist, the mechanism behind an increased susceptibility to epileptic spasms in patients with Down syndrome has not yet been fully elucidated and remains an area for further study. *GIRK2* has been identified as a potential contributor to epileptic spasms through studies of the Ts65Dn mouse model, but overexpression of *GIRK2* alone has been shown to be insufficient for the development of epileptic spasms. Additionally, genetic studies of individuals with DS and epileptic spasms have not shown any genetic variants present in more than one or two participants. Better understanding of the role of genes in causing epileptic spasms could potentially aid in the diagnosis and treatment of this condition, not only in patients with Down syndrome, but in the population at large.

This review summarizes results from a relatively large cohort of individuals with epileptic spasms and DS, but most studies were retrospective analyses, and results, including lifetime prevalence data, treatment response, and developmental outcomes, were still variable among studies. As epileptic spasms are rare, and individuals with Down syndrome and epileptic spasms make up a small proportion of the total population of individuals with DS, multicenter prospective studies will be necessary to better characterize the natural history of this disorder and to improve both treatment and outcomes.

## CONFLICTS OF INTEREST

BGS occasionally consults on the topic of Down syndrome through Gerson Lehrman Group. He receives remuneration from Down syndrome nonprofit organizations for speaking engagements and associated travel expenses. BGS receives annual royalties from Woodbine House, Inc, for the publication of his book, *Fasten Your Seatbelt: A Crash Course on Down Syndrome for Brothers and Sisters*. Within the past two years, he has received research funding from F. Hoffmann‐La Roche, Inc and LuMind IDSC Research Down Syndrome Foundation to conduct clinical trials for people with Down syndrome. BGS is occasionally asked to serve as an expert witness for legal cases where Down syndrome is discussed. BGS serves in a nonpaid capacity on the Honorary Board of Directors for the Massachusetts Down Syndrome Congress and the Professional Advisory Committee for the National Center for Prenatal and Postnatal Down Syndrome Resources. BGS has a sister with Down syndrome. The remaining authors have no conflicts of interest. We confirm that we have read the Journal's position on issues involved in ethical publication and affirm that this report is consistent with those guidelines.

## AUTHOR CONTRIBUTIONS

KJR and BGS contributed to the conception and design of this work. KJR and DJK contributed to the literature review as well as the drafting of the manuscript and figures. All authors contributed to the editing of the manuscript.
